# *N*-(2,3-Dihydro-1,4-benzodioxin-6-yl)-4-fluoro­benzene­sulfonamide

**DOI:** 10.1107/S1600536812030863

**Published:** 2012-07-10

**Authors:** Shumaila Younas Mughal, Islam Ullah Khan, William T. A. Harrison, Muneeb Hayat Khan, Muhammad Nadeem Arshad

**Affiliations:** a Materials Chemistry Laboratory, Department of Chemistry, GC University, Lahore 54000, Pakistan; b Department of Chemistry, University of Aberdeen, Meston Walk, Aberdeen AB24 3UE, Scotland; c Questioned Documents Unit, Punjab Forensic Science Agency, Home Department, Lahore, Pakistan; d The Center of Excellence for Advanced Materials Research (CEAMR), Faculty of Science, King Abdulaziz University, PO Box 80203, Jeddah 21589, Saudi Arabia

## Abstract

In the title compound, C_14_H_12_FNO_4_S, the dihedral angle between the aromatic rings is 50.26 (9)° and the C—S—N—C bond adopts a *gauche* conformation [torsion angle = −68.12 (15)°]. The dihydro­dioxine ring is disordered over two orientations, which both approximate to half-chairs, in a 0.880 (7):0.120 (7) ratio. In the crystal, N—H⋯O hydrogen bonds link the mol­ecules into *C*(4) chains propagating in [100]. Weak C—H⋯O and C—H⋯F inter­actions consolidate the packing.

## Related literature

For related structures, see: Khan *et al.* (2011 ▶); Gelbrich *et al.* (2007 ▶).
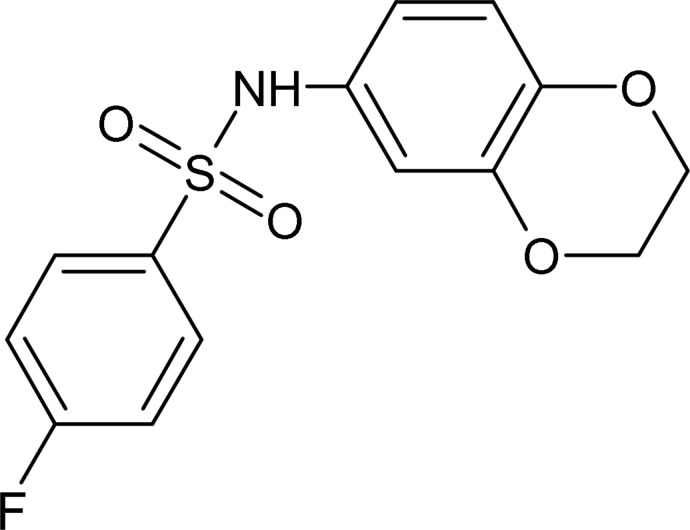


## Experimental

### 

#### Crystal data

C_14_H_12_FNO_4_S
*M* *_r_* = 309.31
Monoclinic, 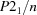

*a* = 5.1542 (5) Å
*b* = 22.237 (3) Å
*c* = 12.0706 (13) Å
β = 94.422 (3)°
*V* = 1379.3 (3) Å^3^
*Z* = 4
Mo *K*α radiation
μ = 0.26 mm^−1^
*T* = 296 K
0.39 × 0.34 × 0.23 mm

#### Data collection

Bruker APEXII CCD diffractometer
11762 measured reflections
3156 independent reflections
2336 reflections with *I* > 2σ(*I*)
*R*_int_ = 0.024

#### Refinement

*R*[*F* ^2^ > 2σ(*F* ^2^)] = 0.037
*wR*(*F* ^2^) = 0.101
*S* = 1.02
3156 reflections
202 parameters
H atoms treated by a mixture of independent and constrained refinement
Δρ_max_ = 0.24 e Å^−3^
Δρ_min_ = −0.32 e Å^−3^

### 

Data collection: *APEX2* (Bruker, 2007 ▶); cell refinement: *SAINT* (Bruker, 2007 ▶); data reduction: *SAINT*; program(s) used to solve structure: *SHELXS97* (Sheldrick, 2008 ▶); program(s) used to refine structure: *SHELXL97* (Sheldrick, 2008 ▶); molecular graphics: *ORTEP-3* (Farrugia, 1997 ▶); software used to prepare material for publication: *SHELXL97*.

## Supplementary Material

Crystal structure: contains datablock(s) global, I. DOI: 10.1107/S1600536812030863/bt5970sup1.cif

Structure factors: contains datablock(s) I. DOI: 10.1107/S1600536812030863/bt5970Isup2.hkl

Supplementary material file. DOI: 10.1107/S1600536812030863/bt5970Isup3.cml

Additional supplementary materials:  crystallographic information; 3D view; checkCIF report

## Figures and Tables

**Table 1:** Hydrogen-bond geometry (Å, °)

*D*—H⋯*A*	*D*—H	H⋯*A*	*D*⋯*A*	*D*—H⋯*A*
N1—H1⋯O2^i^	0.81 (2)	2.22 (2)	3.009 (2)	164 (2)
C3—H3⋯O1^ii^	0.93	2.53	3.368 (3)	150
C5—H5⋯O4^iii^	0.93	2.56	3.391 (2)	149
C8—H8⋯O3^iv^	0.93	2.52	3.446 (2)	172
C13—H13*B*⋯F1^v^	0.97	2.48	3.129 (3)	125

Symmetry codes: (i) 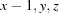; (ii) 
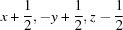
; (iii) 
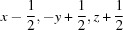
; (iv) 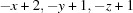; (v) 
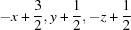
.

**Fig. 1.**
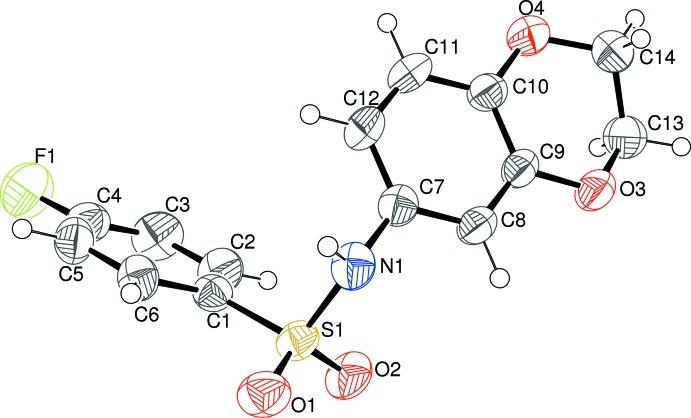
The molecular structure of (I), showing displacement ellipsoids at the 50% probability level. Only the major disorder component is shown.

**Fig. 2.**
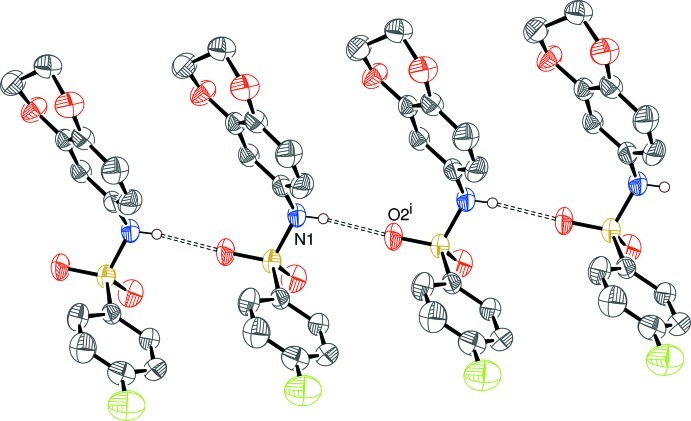
The partial packing diagram for (I) showing the formation of C(4) chains: hydrogen bonds are shown as double-dashed lines and all C-bound H atoms are omitted for clarity. Symmetry code: (i) x–1, y, z.

## supplementary crystallographic information

### Comment

The title compound, (I), (Fig. 1) was examined as part of our ongoing interest in the structural chemistry of sulfonamides (Khan *et al.*, 2011). A number of related structures have been reported by Gelbrich *et al.* (2007).

The dihedral angle between the C1—C6 and C7—C12 benzene rings in (I) is 50.26 (9)°. The C1—S1—N1—C7 linkage adopts a *gauche* conformation [torsion angle = -68.12 (15)°] and the bond-angle sum about N1 (H atom coordinates freely refined) is 347.2°, possibly suggesting a hybridization state intermediate between *sp*^2^ and *sp*^3^. The largest bond angle at the distorted tetrahedral S atom is O1—S1—O2 [120.26 (9)°], which is typical for this class of compound (Khan *et al.*, 2011).

Atoms C13 and C14 and their attached H atoms of the dihydro-dioxin ring are disordered over two sets of sites in a 0.880 (7):0.120 (7) ratio. Both major and minor conformations approximate to a half-chair. In the major conformation, C13 and C14 are displaced from the plane defined by C7—C12/O3/O4 (r.m.s. deviation = 0.037 Å) by 0.212 (3) and -0.556 (3) Å, respectively. The equivalent atoms in the minor component are displaced by -0.44 (3) and 0.41 (2) Å, respectively.

In the crystal, the molecules are linked by N—H···O hydrogen bonds (Table 1) to generate C(4) chains propagating in [100] (Figure 2). Weak C—H···O and C—H···F interactions also occur but there is no aromatic π-π stacking in the structure of (I).

### Experimental

0.2 g of 6-amino 1,4-benzodioxan was dissolved in 15 ml dichloromethane and 0.25 g of 4-fluorobenzene sulfonyl chloride was added to the mixture, which was stirred at room temperature overnight. The pH was maintained at 8–9 with triethyamine. On completion of reaction (after TLC) the pH was adjusted to 1–2 with 1 *M* HCl solution. The organic fraction was collected and the solvent was allowed to evaporate at room temperature. Colourless prisms of (I) were obtained in 95% yield.

### Refinement

The N-bond H atom was located in a difference map and its position was freely refined. The C-bound H atoms were placed in calculated positions (C—H = 0.93–0.97 Å) and refined as riding. The constraint *U*_iso_(H) = 1.2*U*_eq_(C,N) was applied.

### Crystal data

**Table:**

C_14_H_12_FNO_4_S	*F*(000) = 640
*M**_r_* = 309.31	*D*_x_ = 1.489 Mg m^−^^3^
Monoclinic, *P*2_1_/*n*	Mo *K*α radiation, λ = 0.71073 Å
*a* = 5.1542 (5) Å	Cell parameters from 1453 reflections
*b* = 22.237 (3) Å	θ = 2.5–25.5°
*c* = 12.0706 (13) Å	µ = 0.26 mm^−^^1^
β = 94.422 (3)°	*T* = 296 K
*V* = 1379.3 (3) Å^3^	Prism, colourless
*Z* = 4	0.39 × 0.34 × 0.23 mm

### Data collection

**Table:**

Bruker APEXII CCD diffractometer	2336 reflections with *I* > 2σ(*I*)
Radiation source: fine-focus sealed tube	*R*_int_ = 0.024
Graphite monochromator	θ_max_ = 27.5°, θ_min_ = 1.8°
ω scans	*h* = −6→5
11762 measured reflections	*k* = −28→28
3156 independent reflections	*l* = −15→14

### Refinement

**Table:**

Refinement on *F*^2^	Primary atom site location: structure-invariant direct methods
Least-squares matrix: full	Secondary atom site location: difference Fourier map
*R*[*F*^2^ > 2σ(*F*^2^)] = 0.037	Hydrogen site location: inferred from neighbouring sites
*wR*(*F*^2^) = 0.101	H atoms treated by a mixture of independent and constrained refinement
*S* = 1.02	*w* = 1/[σ^2^(*F*_o_^2^) + (0.0451*P*)^2^ + 0.3224*P*] where *P* = (*F*_o_^2^ + 2*F*_c_^2^)/3
3156 reflections	(Δ/σ)_max_ < 0.001
202 parameters	Δρ_max_ = 0.24 e Å^−^^3^
0 restraints	Δρ_min_ = −0.32 e Å^−^^3^

### Special details

**Table:**

Geometry. All e.s.d.'s (except the e.s.d. in the dihedral angle between two l.s. planes) are estimated using the full covariance matrix. The cell e.s.d.'s are taken into account individually in the estimation of e.s.d.'s in distances, angles and torsion angles; correlations between e.s.d.'s in cell parameters are only used when they are defined by crystal symmetry. An approximate (isotropic) treatment of cell e.s.d.'s is used for estimating e.s.d.'s involving l.s. planes.
Refinement. Refinement of *F*^2^ against ALL reflections. The weighted *R*-factor *wR* and goodness of fit *S* are based on *F*^2^, conventional *R*-factors *R* are based on *F*, with *F* set to zero for negative *F*^2^. The threshold expression of *F*^2^ > σ(*F*^2^) is used only for calculating *R*-factors(gt) *etc*. and is not relevant to the choice of reflections for refinement. *R*-factors based on *F*^2^ are statistically about twice as large as those based on *F*, and *R*- factors based on ALL data will be even larger.

### Fractional atomic coordinates and isotropic or equivalent isotropic displacement parameters (Å^2^)

**Table:**

	*x*	*y*	*z*	*U*_iso_*/*U*_eq_	Occ. (<1)
C1	0.5881 (3)	0.25665 (7)	0.60034 (14)	0.0404 (4)	
C2	0.7189 (4)	0.23608 (9)	0.51210 (17)	0.0565 (5)	
H2	0.8515	0.2590	0.4852	0.068*	
C3	0.6514 (5)	0.18133 (10)	0.46402 (18)	0.0664 (6)	
H3	0.7375	0.1668	0.4046	0.080*	
C4	0.4572 (4)	0.14930 (9)	0.50536 (17)	0.0564 (5)	
C5	0.3224 (4)	0.16869 (8)	0.59056 (17)	0.0543 (5)	
H5	0.1876	0.1458	0.6154	0.065*	
C6	0.3891 (4)	0.22311 (8)	0.63959 (15)	0.0482 (4)	
H6	0.3006	0.2371	0.6987	0.058*	
F1	0.3949 (3)	0.09489 (6)	0.45976 (12)	0.0886 (4)	
C7	0.4989 (3)	0.39470 (7)	0.48965 (15)	0.0429 (4)	
C8	0.6750 (3)	0.43897 (7)	0.46691 (15)	0.0434 (4)	
H8	0.7859	0.4549	0.5239	0.052*	
C9	0.6860 (3)	0.45950 (7)	0.35926 (14)	0.0415 (4)	
C10	0.5168 (3)	0.43651 (8)	0.27495 (15)	0.0457 (4)	
C11	0.3469 (4)	0.39124 (9)	0.29867 (18)	0.0613 (5)	
H11	0.2378	0.3746	0.2417	0.074*	
C12	0.3366 (4)	0.37037 (9)	0.40506 (18)	0.0583 (5)	
H12	0.2208	0.3399	0.4202	0.070*	
C13	0.8923 (7)	0.51508 (15)	0.2265 (2)	0.0598 (8)	0.880 (7)
H13A	1.0029	0.4842	0.1985	0.072*	0.880 (7)
H13B	0.9770	0.5536	0.2185	0.072*	0.880 (7)
C13A	0.794 (6)	0.5341 (11)	0.2265 (19)	0.064 (7)*	0.120 (7)
H13C	0.6299	0.5555	0.2269	0.076*	0.120 (7)
H13D	0.9283	0.5628	0.2111	0.076*	0.120 (7)
C14	0.6350 (6)	0.51509 (11)	0.1600 (2)	0.0578 (9)	0.880 (7)
H14A	0.5239	0.5459	0.1879	0.069*	0.880 (7)
H14B	0.6601	0.5241	0.0830	0.069*	0.880 (7)
C14A	0.775 (4)	0.4882 (9)	0.1419 (15)	0.058 (6)*	0.120 (7)
H14C	0.9199	0.4603	0.1504	0.070*	0.120 (7)
H14D	0.7654	0.5054	0.0679	0.070*	0.120 (7)
S1	0.66702 (8)	0.32659 (2)	0.66316 (4)	0.04491 (14)	
N1	0.4773 (3)	0.37724 (7)	0.60361 (14)	0.0490 (4)	
H1	0.330 (4)	0.3731 (9)	0.6218 (16)	0.059*	
O1	0.5996 (3)	0.32377 (7)	0.77539 (11)	0.0620 (4)	
O2	0.9274 (2)	0.34095 (6)	0.63905 (12)	0.0603 (4)	
O3	0.8603 (2)	0.50425 (6)	0.34055 (10)	0.0564 (4)	
O4	0.5128 (3)	0.45743 (6)	0.16736 (11)	0.0586 (4)	

### Atomic displacement parameters (Å^2^)

**Table:**

	*U*^11^	*U*^22^	*U*^33^	*U*^12^	*U*^13^	*U*^23^
C1	0.0334 (9)	0.0456 (9)	0.0420 (9)	0.0026 (7)	0.0025 (7)	0.0036 (7)
C2	0.0493 (12)	0.0695 (12)	0.0524 (11)	−0.0057 (9)	0.0153 (9)	−0.0025 (9)
C3	0.0714 (15)	0.0770 (14)	0.0526 (12)	0.0018 (11)	0.0160 (10)	−0.0162 (10)
C4	0.0634 (14)	0.0503 (10)	0.0537 (12)	0.0027 (9)	−0.0074 (10)	−0.0055 (9)
C5	0.0500 (12)	0.0474 (10)	0.0659 (13)	−0.0054 (8)	0.0062 (9)	0.0070 (9)
C6	0.0437 (11)	0.0485 (9)	0.0536 (11)	0.0015 (8)	0.0120 (8)	0.0043 (8)
F1	0.1154 (12)	0.0617 (8)	0.0870 (10)	−0.0070 (7)	−0.0023 (8)	−0.0230 (7)
C7	0.0333 (9)	0.0413 (8)	0.0543 (11)	0.0025 (7)	0.0050 (7)	−0.0014 (7)
C8	0.0368 (9)	0.0424 (9)	0.0500 (10)	−0.0032 (7)	−0.0025 (7)	−0.0069 (7)
C9	0.0337 (9)	0.0372 (8)	0.0530 (10)	−0.0017 (6)	0.0007 (7)	−0.0061 (7)
C10	0.0435 (10)	0.0427 (9)	0.0500 (11)	0.0015 (7)	−0.0034 (8)	−0.0053 (7)
C11	0.0547 (13)	0.0606 (12)	0.0653 (13)	−0.0182 (9)	−0.0154 (10)	−0.0056 (10)
C12	0.0457 (11)	0.0553 (11)	0.0725 (14)	−0.0174 (9)	−0.0049 (9)	0.0033 (10)
C13	0.0550 (18)	0.0660 (17)	0.0580 (16)	−0.0102 (14)	0.0026 (12)	0.0107 (12)
C14	0.0650 (19)	0.0518 (13)	0.0555 (15)	−0.0002 (11)	−0.0029 (12)	0.0041 (10)
S1	0.0329 (2)	0.0524 (3)	0.0494 (3)	−0.00419 (18)	0.00263 (17)	−0.00209 (19)
N1	0.0342 (8)	0.0510 (8)	0.0630 (10)	0.0015 (7)	0.0122 (7)	0.0001 (7)
O1	0.0648 (9)	0.0763 (9)	0.0449 (8)	−0.0063 (7)	0.0039 (6)	−0.0058 (6)
O2	0.0297 (7)	0.0679 (9)	0.0833 (10)	−0.0074 (6)	0.0038 (6)	−0.0003 (7)
O3	0.0567 (9)	0.0584 (8)	0.0529 (8)	−0.0218 (6)	−0.0032 (6)	0.0030 (6)
O4	0.0665 (9)	0.0568 (8)	0.0505 (8)	−0.0093 (6)	−0.0090 (6)	−0.0024 (6)

### Geometric parameters (Å, º)

**Table:**

C1—C6	1.381 (2)	C11—C12	1.370 (3)
C1—C2	1.381 (2)	C11—H11	0.9300
C1—S1	1.7640 (17)	C12—H12	0.9300
C2—C3	1.382 (3)	C13—O3	1.419 (3)
C2—H2	0.9300	C13—C14	1.496 (4)
C3—C4	1.355 (3)	C13—H13A	0.9700
C3—H3	0.9300	C13—H13B	0.9700
C4—C5	1.355 (3)	C13A—C14A	1.44 (3)
C4—F1	1.357 (2)	C13A—O3	1.54 (2)
C5—C6	1.379 (3)	C13A—H13C	0.9700
C5—H5	0.9300	C13A—H13D	0.9700
C6—H6	0.9300	C14—O4	1.434 (3)
C7—C12	1.379 (2)	C14—H14A	0.9700
C7—C8	1.381 (2)	C14—H14B	0.9700
C7—N1	1.442 (2)	C14A—O4	1.566 (18)
C8—C9	1.383 (2)	C14A—H14C	0.9700
C8—H8	0.9300	C14A—H14D	0.9700
C9—O3	1.371 (2)	S1—O1	1.4256 (14)
C9—C10	1.386 (2)	S1—O2	1.4312 (13)
C10—O4	1.378 (2)	S1—N1	1.6228 (16)
C10—C11	1.379 (3)	N1—H1	0.81 (2)
			
C6—C1—C2	120.33 (17)	O3—C13—H13A	109.5
C6—C1—S1	118.77 (13)	C14—C13—H13A	109.5
C2—C1—S1	120.88 (14)	O3—C13—H13B	109.5
C1—C2—C3	119.58 (19)	C14—C13—H13B	109.5
C1—C2—H2	120.2	H13A—C13—H13B	108.1
C3—C2—H2	120.2	C14A—C13A—O3	109.0 (18)
C4—C3—C2	118.54 (19)	C14A—C13A—H13C	109.9
C4—C3—H3	120.7	O3—C13A—H13C	109.9
C2—C3—H3	120.7	C14A—C13A—H13D	109.9
C3—C4—C5	123.30 (19)	O3—C13A—H13D	109.9
C3—C4—F1	118.53 (19)	H13C—C13A—H13D	108.3
C5—C4—F1	118.17 (19)	O4—C14—C13	110.0 (2)
C4—C5—C6	118.64 (18)	O4—C14—H14A	109.7
C4—C5—H5	120.7	C13—C14—H14A	109.7
C6—C5—H5	120.7	O4—C14—H14B	109.7
C5—C6—C1	119.59 (17)	C13—C14—H14B	109.7
C5—C6—H6	120.2	H14A—C14—H14B	108.2
C1—C6—H6	120.2	C13A—C14A—O4	100.5 (18)
C12—C7—C8	120.18 (17)	C13A—C14A—H14C	111.7
C12—C7—N1	120.90 (16)	O4—C14A—H14C	111.7
C8—C7—N1	118.81 (16)	C13A—C14A—H14D	111.7
C7—C8—C9	119.83 (16)	O4—C14A—H14D	111.7
C7—C8—H8	120.1	H14C—C14A—H14D	109.4
C9—C8—H8	120.1	O1—S1—O2	120.26 (9)
O3—C9—C8	117.99 (14)	O1—S1—N1	105.59 (9)
O3—C9—C10	122.01 (16)	O2—S1—N1	107.32 (9)
C8—C9—C10	119.95 (16)	O1—S1—C1	107.74 (8)
O4—C10—C11	118.58 (16)	O2—S1—C1	107.34 (8)
O4—C10—C9	122.02 (16)	N1—S1—C1	108.09 (8)
C11—C10—C9	119.39 (17)	C7—N1—S1	121.42 (12)
C12—C11—C10	120.85 (17)	C7—N1—H1	115.5 (14)
C12—C11—H11	119.6	S1—N1—H1	110.2 (15)
C10—C11—H11	119.6	C9—O3—C13	114.18 (15)
C11—C12—C7	119.74 (17)	C9—O3—C13A	110.9 (10)
C11—C12—H12	120.1	C10—O4—C14	112.66 (14)
C7—C12—H12	120.1	C10—O4—C14A	112.4 (7)
O3—C13—C14	110.8 (2)		
			
C6—C1—C2—C3	−0.8 (3)	C2—C1—S1—O1	−153.38 (16)
S1—C1—C2—C3	−179.50 (16)	C6—C1—S1—O2	158.72 (14)
C1—C2—C3—C4	0.0 (3)	C2—C1—S1—O2	−22.52 (18)
C2—C3—C4—C5	1.1 (3)	C6—C1—S1—N1	−85.80 (15)
C2—C3—C4—F1	−178.54 (19)	C2—C1—S1—N1	92.95 (16)
C3—C4—C5—C6	−1.5 (3)	C12—C7—N1—S1	97.69 (19)
F1—C4—C5—C6	178.17 (17)	C8—C7—N1—S1	−86.06 (19)
C4—C5—C6—C1	0.7 (3)	O1—S1—N1—C7	176.79 (14)
C2—C1—C6—C5	0.4 (3)	O2—S1—N1—C7	47.37 (16)
S1—C1—C6—C5	179.15 (14)	C1—S1—N1—C7	−68.12 (15)
C12—C7—C8—C9	0.9 (3)	C8—C9—O3—C13	169.3 (2)
N1—C7—C8—C9	−175.37 (15)	C10—C9—O3—C13	−13.3 (3)
C7—C8—C9—O3	178.83 (15)	C8—C9—O3—C13A	−163.5 (11)
C7—C8—C9—C10	1.3 (3)	C10—C9—O3—C13A	13.9 (11)
O3—C9—C10—O4	−0.3 (3)	C14—C13—O3—C9	43.1 (3)
C8—C9—C10—O4	177.12 (15)	C14—C13—O3—C13A	−45 (2)
O3—C9—C10—C11	179.60 (17)	C14A—C13A—O3—C9	−54 (2)
C8—C9—C10—C11	−3.0 (3)	C14A—C13A—O3—C13	48.9 (19)
O4—C10—C11—C12	−177.64 (18)	C11—C10—O4—C14	161.9 (2)
C9—C10—C11—C12	2.5 (3)	C9—C10—O4—C14	−18.2 (3)
C10—C11—C12—C7	−0.3 (3)	C11—C10—O4—C14A	−157.3 (9)
C8—C7—C12—C11	−1.5 (3)	C9—C10—O4—C14A	22.6 (9)
N1—C7—C12—C11	174.74 (18)	C13—C14—O4—C10	47.5 (3)
O3—C13—C14—O4	−61.6 (4)	C13—C14—O4—C14A	−50.3 (11)
O3—C13A—C14A—O4	72 (2)	C13A—C14A—O4—C10	−57.2 (18)
C6—C1—S1—O1	27.86 (16)	C13A—C14A—O4—C14	41.3 (14)

### Hydrogen-bond geometry (Å, º)

**Table:**

*D*—H···*A*	*D*—H	H···*A*	*D*···*A*	*D*—H···*A*
N1—H1···O2^i^	0.81 (2)	2.22 (2)	3.009 (2)	164 (2)
C3—H3···O1^ii^	0.93	2.53	3.368 (3)	150
C5—H5···O4^iii^	0.93	2.56	3.391 (2)	149
C8—H8···O3^iv^	0.93	2.52	3.446 (2)	172
C13—H13*B*···F1^v^	0.97	2.48	3.129 (3)	125

Symmetry codes: (i) *x*−1, *y*, *z*; (ii) *x*+1/2, −*y*+1/2, *z*−1/2; (iii) *x*−1/2, −*y*+1/2, *z*+1/2; (iv) −*x*+2, −*y*+1, −*z*+1; (v) −*x*+3/2, *y*+1/2, −*z*+1/2.
